# Factors Associated with COVID-19 Vaccine Uptake among Schoolgoing Adolescent Girls and Young Women in South Africa

**DOI:** 10.3390/vaccines11101581

**Published:** 2023-10-11

**Authors:** Kate Bergh, Kim Jonas, Zoe Duby, Darshini Govindasamy, Catherine Mathews, Tarylee Reddy, Nevilene Slingers, Granville Whittle, Fareed Abdullah

**Affiliations:** 1 Health Systems Research Unit, South African Medical Research Council, Cape Town 7501, South Africa; kim.jonas@mrc.ac.za (K.J.); zoe.duby@mrc.ac.za (Z.D.); darshini.govindasamy@mrc.ac.za (D.G.); catherine.mathews@mrc.ac.za (C.M.); 2 Department of Psychology, University of Cape Town, Cape Town 7700, South Africa; 3 Division of Social and Behavioural Sciences, School of Public Health and Family Medicine, University of Cape Town, Cape Town 7925, South Africa; 4 School of Public Health and Family Medicine, University of Cape Town, Cape Town 7925, South Africa; 5 Biostatistics Research Unit, South African Medical Research Council, Durban 4091, South Africa; tarylee.reddy@mrc.ac.za; 6 Office of AIDS and TB Research, South African Medical Research Council, Pretoria 0001, South Africa; nevilene.slingers@mrc.ac.za (N.S.); fareed.abdullah@mrc.ac.za (F.A.); 7 Department of Basic Education, Government of South Africa, Pretoria 0001, South Africa; 8 Division of Infectious Diseases, Department of Internal Medicine, Steve Biko Academic Hospital and University of Pretoria, Pretoria 0002, South Africa

**Keywords:** COVID-19 vaccination, prevention cascades, adolescent girls and young women, South Africa

## Abstract

(1) Background: By October 2022, vaccination rates with at least one dose of a COVID-19 vaccine were low among adolescent girls aged 12–17 (38%) and young women aged 18–34 (45%) in South Africa. This study aimed to measure and identify barriers to and facilitators of motivation to take up, access to, and uptake of COVID-19 vaccines among schoolgoing adolescent girls and young women in two districts in South Africa. (2) Methods: Using the theory of the HIV prevention cascade, we conceptualised the relationship between motivation, access, and uptake of COVID-19 vaccines, and associated barriers. Potential barriers and facilitators were identified using bivariate and multivariable Poisson regression. (3) Results: Among all 2375 participants, access was high (69%), but motivation (49%) and vaccination with at least one COVID-19 vaccine (45%) were lower. Fear of injections was a barrier to vaccine uptake (aRR 0.85 95% CI 0.82–0.88), while being tested for COVID-19 (aRR 2.10 95% CI 1.85–2.38) and believing that the COVID-19 vaccine was safe (aRR 1.31 95% CI 1.18–1.44) and would prevent you from getting very sick (aRR 1.11 95% CI 1.04–1.19) were facilitators. (4) Conclusions: The controversy about the value of vaccinating adolescents and the delay in vaccine rollout for adolescents and young adults may have contributed to fears about the safety and efficacy of COVID-19 vaccines, as well as a lack of motivation to get vaccinated.

## 1. Background

By September 2023, it had been three and a half years since the World Health Organisation (WHO) declared that severe acute respiratory syndrome coronavirus 2 (SARS-CoV-2) was a public health emergency of international concern [1]. By this time, there had been almost seven million reported deaths due to SARS-CoV-2 worldwide [2]. South Africa had reported a total of 102,595 SARS-CoV-2 related deaths, although recent data on excess deaths suggest that this figure could be over three times higher [3].

The South African government implemented a number of interventions to stop the spread of COVID-19 over the course of the pandemic, including a national lockdown, social distancing, and mask wearing in public. Although the lockdown may have slowed the initial spread of COVID-19, it had devastating impacts on household financial security, prevention and treatment of other communicable diseases, educational outcomes, and mental health [4,5,6,7]. Adolescent girls and young women (AGYW) were especially vulnerable to HIV infection, unintended pregnancies, educational disruptions, stress, and anxiety during the national lockdown, which may have lasting effects. 

In terms of COVID-19 cases among younger people, most COVID-19 cases among children and adolescents under 19 years ranged from asymptomatic to mild-to-moderate disease in South Africa and internationally during the first three waves of the pandemic [8]. However, the emergence of the Omicron variant of SARS-CoV-2 (the virus that causes the COVID-19 disease) during the fourth wave of COVID-19 in South Africa (November–December 2021) saw an increased number of hospitalisations among this sub-population. This may be due to increased transmissibility of the virus and lower COVID-19 vaccination rates among children and adolescents under 18 years. 

In South Africa, two COVID-19 vaccines (one dose of the Janssen^®^ (J&J) vaccine or two doses of Comirnaty^®^ (Pfizer)) were approved for use by young adults aged 18–34 from September 2021 [9]. Adolescents aged 12–17 were eligible for one dose of the Pfizer vaccine from October 2021 and two doses from December 2021 [10]. By October 2022 (the end of data collection for this study), 30.2% and 37.8% of adolescent boys and girls, respectively, had received one dose of the Pfizer vaccine [11]. In the 18–34 age group, 34.4% of men and 44.6% of women had received one dose of the J&J or Pfizer vaccine. 

Vaccination rates in South Africa are low compared to other Eastern and Southern African countries (15th out of 20) [12]. Given that adolescents and young people aged 10–24 make up approximately one quarter of South Africa’s population, vaccinating this sub-population is critical to reducing transmission in the total population and reducing severe disease and death among young people in the country [13]. Vaccination with two doses of the Pfizer vaccine has been shown to reduce COVID-19-associated hospitalisations among adolescents aged 12–18 by 94% in the United States [14].

Despite the many benefits of COVID-19 vaccination, ‘vaccine hesitancy’ in South Africa is as high as 32% [15]. Vaccine hesitancy is defined as a delay in acceptance or refusal to get vaccinated despite the availability of vaccination services [16]. Several studies conducted among adults in South Africa before October 2021 have found that vaccine hesitancy was associated with younger age, higher levels of education, being male, having no monthly income, living outside of urban areas, having no access to the online vaccination registration portal, not believing that the government was capable of delivering safe and effective vaccines, conspiracy theories, being worried about side effects, being worried about the safety and effectiveness of COVID-19 vaccines, low COVID-19 risk perception, and depending on someone else for vaccination decision [9,15,17]. 

While there is limited literature on the reasons for vaccine hesitancy among adolescents in South Africa, a study conducted between July and December 2021 among 2662 adolescents aged 10–19 across five sub-Saharan African countries (excluding South Africa) found that the key factors associated with vaccine hesitancy were perceived low necessity and concerns about safety and effectiveness [18]. In South Africa, a study conducted in May 2021 asked 16 young people aged 17–29 on Facebook an open-ended question about whether they would receive the COVID-19 vaccine if it was made available to them [19]. Findings were similar to those among adults in South Africa and adolescents in sub-Saharan Africa, but also included fear of injections and a preference for traditional remedies, while reasons for vaccine acceptance included receiving information from trusted sources and observing safe vaccine uptake among people they trusted [19]. The barriers to, and facilitators of vaccine uptake among adolescents in South Africa and sub-Saharan Africa are very similar to those described by a global systematic review of quantitative studies on the topic [20].

Although vaccine hesitancy in South Africa is high, access to vaccination services may also be an obstacle to vaccine uptake, which is poorly documented in the literature. However, access to health services is a major barrier to uptake of and adherence to other prevention methods, including those for HIV and family planning [21,22]. While HIV prevention methods, family planning, and COVID-19 vaccines are all provided for free through South Africa’s public health sector, HIV prevention methods and family planning are provided through government primary care facilities, and COVID-19 vaccines were provided at newly established vaccination sites throughout South Africa, including in schools. While schools may have been used as vaccination sites, learners were not necessarily encouraged to get vaccinated, as there was no vaccination promotional or educational programme rolled out by the government in school, but demand creation through television, radio, and posters was common.

This study will follow the logic of the HIV prevention cascade (the unifying framework) to measure motivation, access, and uptake of COVID-19 vaccines, as well as identify barriers to and facilitators of each step among AGYW in 14 high schools across two districts in South Africa who participated in a process and outcomes evaluation [23,24,25]. The HIV prevention cascade is a novel framework that incorporates both behavioural, social and structural theories of behaviour change, providing a comprehensive model of the steps required to take up a disease prevention method [26]. This conceptualisation of the HIV prevention cascade has been chosen because it is the most appropriate for low-resource settings, as it does not require access to sophisticated data systems and mathematical modelling to construct, thus allowing for comparison with other studies in this region in the future. The findings will help to inform targeted interventions to improve vaccine uptake in the event of COVID-19 surges due to new variants or future national or global outbreaks of new pathogen pandemics.

## 2. Methods and Materials

### 2.1. Study Setting

This study examines motivation, access, and uptake of COVID-19 vaccines among AGYW using data from the baseline survey of a process and outcomes evaluation (Imagine Evaluation) of a combination HIV prevention programme (The Imagine Programme). The survey was conducted in July–September 2022 by the Health Systems Research Unit at the South African Medical Research Council (SAMRC). The Imagine Programme was developed by the SAMRC’s Office of AIDS and TB Research, NACOSA, and other stakeholders, and is funded through a novel social impact bond [27]. The programme started in June 2023, after the baseline survey.

The Imagine Programme will be rolled out in 6 schools in the Moretele area of the North-West Province, and 8 schools in the Newcastle area of Kwazulu-Natal Province. Moretele is a rural site located one-hour from Johannesburg, while Newcastle is a semi-urban area with the closest major city, Johannesburg, more than a three-hour drive away [28,29]. Both study areas have high rates of poverty and unemployment, HIV infection, and teenage pregnancy [28,29]. Although the primary focus of the programme is pregnancy and HIV prevention and care, the programme seeks to improve the overall health and well-being of its beneficiaries by offering a wide range of services, including clinical sexual and reproductive health services, psycho-social support services, and social structural services. The Imagine Evaluation assessed COVID-19 testing, deaths, and vaccination among participants to understand the impact of the COVID-19 pandemic on participant health and well-being to ensure that the programme provides appropriate services to its beneficiaries.

This data provides valuable information on vaccination rates, the barriers to, and facilitators of COVID-19 vaccines among vulnerable and hard-to-reach communities in South Africa. Since schools are the ideal environment to roll out vaccination programmes, these data provide useful information from the perspective of schoolgoing AGYW. Although COVID-19 vaccination was lower among adolescent boys and young men, our data only focuses on AGYW given the primary outcomes of the Imagine Programme. Nevertheless, we will compare our results to the literature to see if our findings are similar to those for both boys and girls.

### 2.2. Sample and Data Collection

For the baseline survey, a cross-sectional survey was conducted among AGYW aged 13 and older in grades 9–12 in all 14 programme schools in Moretele and Newcastle. A sample size of 2240 (40 per grade) AGYW across all schools was required to have more than 80% statistical power to measure desired changes in primary outcomes at the follow-up survey based on a pair-matched study design. Two to three classes per grade were randomly sampled for the study to ensure a minimum of 40 participants per grade. A back-up class was also randomly sampled in case one of the sampled classes could not participate.

### 2.3. Ethical Considerations

Ethics approval was granted by the SAMRC’s Research and Ethics Committee (EC045-10/2020). An informed consent process was conducted with all AGYW before participation in the survey. Parental consent was first obtained from a parent or legal guardian for AGYW who were under 18 years old. Participants filled in the survey themselves on a tablet. The survey questions were audio-assisted, and available in English and the predominant languages in the programme areas (isiZulu or Setswana). Participants received R150 reimbursement for their time.

### 2.4. Measures

The COVID-19 prevention cascade measures the proportion of the population in need of COVID-19 vaccines that were motivated to take them up, had access to them, and were partially or fully vaccinated. Definitions for the steps of the COVID-19 prevention cascade, adapted from the HIV prevention cascade [23,24,25], are provided below:

- Motivation: If the COVID-19 vaccines were freely available, AGYW would definitely want to get it
- Access: It is easy or very easy for AGYW to get the COVID-19 vaccine
- Partial or full vaccination: AGYW had at least one dose of a COVID-19 vaccine

The population in need of COVID-19 vaccines in South Africa includes all people aged 12 years and older, thus all participants of the Imagine Evaluation. Definitions for motivation to take up and access to COVID-19 vaccines are based on Moorhouse et al. (2019)’s definitions for the HIV prevention cascade (Moorhouse et al., 2019) [24]. Partial or full vaccination was defined as receiving at least one dose of a COVID-19 vaccine, which is the same definition used by the South African vaccination dashboard, allowing for an easy comparison [11]. In addition, adolescents aged 13–17 years were initially only offered one dose of the vaccine, and thus had less time to be fully vaccinated [10].

Binary and categorical variables relating to socio-demographics, COVID-19 testing and outcomes, as well as potential barriers to, and facilitators of COVID-19 vaccination were created from survey questions and are described in Table 1. A cluster variable with two categories (relatively low and relatively high) was created for socio-economic status (SES) using 13 SES-related variables included in the survey [30]:

1. AGYW was away from home for more than one month in past 12 months
2. Has piped water in household
3. Has flushing toilet in household
4. Household has working electricity
5. Household has a car
6. Household has a computer
7. Household has the internet
8. Household has a refrigerator
9. Household has a stove
10. AGYW or a member of her household went a whole day and night without eating in the past month
11. AGYW has own money
12. AGYW saves money
13. AGYW owes money

The variable was created using the “klaR” package to perform cluster analysis with the K-Modes algorithm [31,32]. Following a review of the literature on COVID-19 vaccine hesitancy, attitudes and beliefs about COVID-19 vaccines were chosen as potential barriers to and facilitators of motivation to use vaccines. Known barriers to accessing health services in South Africa were selected as potential barriers to and facilitators of access to vaccines.

### 2.5. Assumptions

**Figure 1:**
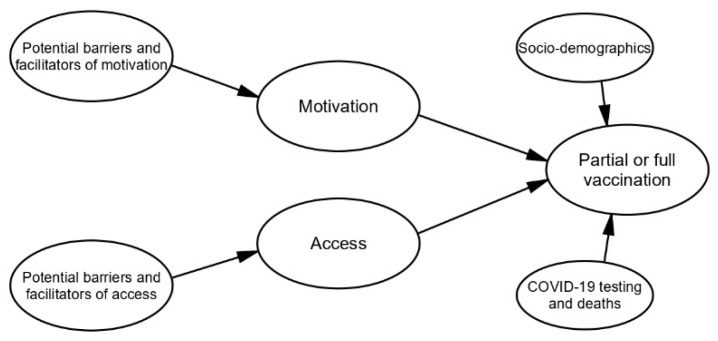
Conceptualisation of the relationship between motivation to take up, access to, partial or full vaccination with, and associated barriers and facilitators of COVID-19 vaccines and vaccination, based on the logic of the HIV prevention cascade [23,24,25].

According to the theory of the HIV prevention cascade (unifying framework), both motivation and access are required for an individual to take up a prevention method [23,24,25]. Figure 1 provides a model of this conceptualisation of the relationship between motivation, access, partial or full vaccination, and associated barriers or facilitators. Socio-demographic factors (age and SES) as well as COVID-19 testing and deaths have been included as potential barriers to, or facilitators of partial or full vaccination, as it is the final outcome in the model.

### 2.6. Statistical Analysis

Statistical analyses were conducted in Stata (Stata SE 17.0, StataCorp, College Station, TX, USA). Participant socio-demographics, COVID-19 testing and outcomes, potential barriers to, and facilitators of COVID-19 vaccine uptake were described for all participants. Unconditional COVID-19 prevention cascades were created for COVID-19 vaccines based on Moorhouse et al. (2019) and Schaeffer et al. (2019)’s methodology [23,24]. All questions had a “prefer not to answer” option which was included in the denominators for these analyses, except in the case of the SES variable where these responses were excluded for clustering.

Poisson regression was used to estimate the relative risk (RR) of motivation to use, access to, and partial or full vaccination for potential barriers to and facilitators of COVID-19 vaccination. For motivation and access, bivariate analyses were conducted with each potential barrier and facilitator individually, adjusting for age and SES group as priori confounders. A final model was then constructed for partial or full vaccination, which included all variables that had a statistically significant relationship with motivation or access (*p* < 0.05), as well as age, SES group, COVID-19 testing, and deaths. The variance inflation factor (VIF) was calculated to test for multicollinearity in the model. A VIF equal to 1 indicates low collinearity, and a VIF equal to 5 indicates high collinearity [33]. “Prefer not to answer” was recoded as missing for the regression analyses. All descriptive and statistical analyses were adjusted for the multistage study design, which included the following stages: (1) schools stratified by district; (2) classes stratified by grade and (3) individual students.

## 3. Results

We enrolled 2377 AGYW participants into our study (sample realisation = 84.0%); 76.0% were in the 13–17 age group (age range: 13–23) (Table 1). In terms of socio-demographics, 39.6% were in the relatively low SES group.

With regards to COVID-19 testing and outcomes, 44.1% of AGYW had been tested for COVID-19, of which 13.7% tested positive (Table 1). Of all participants, 1.7% went to hospital for COVID-19. Among all participants, 26.6% had someone in their home test positive for COVID-19, 17.6% had a family member who went to hospital for COVID-19, 10.4% had a family member who died from COVID-19, and 2.8% had a grandmother who died from COVID-19.

Table 1 also describes the prevalence of potential barriers to, and facilitators of motivation to take up and access to COVID-19 vaccines. Barriers and facilitators that had a statistically significant relationship with vaccine uptake were identified through statistical analyses in Table 2 and Table 3.

**Figure 2:**
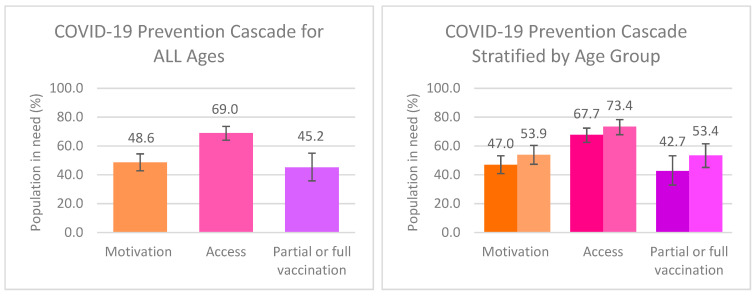
COVID-19 prevention cascades describing motivation, access, and partial or full vaccination of COVID-19 vaccines among ALL school-going participants aged 13–23 (*n* = 2375) and stratified by age group (darker colours = 13–17 age group, *n* = 1815; lighter colours = 18–23 age group, *n* = 560).

Figure 2 describes motivation to take up, access to, and partial or full vaccination with COVID-19 vaccines among all AGYW participants who answered this section of the survey (*n* = 2375); and is stratified by age group. Among all AGYW participants, 48.6% were motivated to take up COVID-19 vaccines, 69.0% had access to these vaccines and 45.2% were partially or fully vaccinated. Only 20.5% of participants reported being fully vaccinated. When stratified by age group, motivation, access, and partial or full vaccination with COVID-19 vaccines were all higher among AGYW aged 18–23 compared to participants aged 13–17, although there was no statistically significant difference between the two age groups.

A bivariate analysis for each potential barrier and facilitator of motivation was conducted, adjusting for age and relative SES (Table 2). All potential barriers and facilitators of motivation had a statistically significant association with motivation and were included in the final model of vaccine uptake (Table 3). The same approach was adopted for potential barriers and facilitators of access. The only factors with a statistically significant association with access and which were included in the final model for vaccine uptake were not knowing where to go to get the vaccine, being expensive to get to the vaccine site, and not being able to go to the vaccine site alone.

Table 3 describes the final multivariable model of all potential barriers to and facilitators of partial or full vaccination with a COVID-19 vaccine. Participants who were very afraid of the vaccine needle were 15% less likely to be partially or fully vaccinated (aRR 0.85 95% CI 0.82–0.88). Participants who had been tested for COVID-19 were more than twice as likely to be partially or fully vaccinated (aRR 2.10 95% CI 1.85–2.38). Participants who agreed that the COVID-19 vaccine would prevent them from getting very sick with COVID-19 and that the vaccine would be safe for them were 11% (aRR 1.11 95% CI 1.04–1.19) and 31% (aRR 1.31 95% CI 1.18–1.44) more likely to be vaccinated, respectively. The VIF for each variable included in the bivariate and final multivariable model was below two, indicating low multicollinearity in the model.

**Table 2:** Bivariate analysis of potential barriers to and facilitators of motivation to take up and access to COVID-19 vaccines, adjusted by age and relative socio-economic status for each individual variable (*n* = 2115).

Variable	aRR	95% CI
**Potential barriers to motivation**		
AGYW * worries that she might get COVID-19 (strongly disagree—strongly agree *)	**1.06**	[1.01,1.12] *
AGYW agrees that COVID-19 would be a serious illness for people in her community (strongly disagree—strongly agree *)	**1.09**	[1.04,1.14] **
AGYW is very afraid of the vaccine needle (strongly disagree—strongly agree *)	**0.87**	[0.85,0.90] **
AGYW agrees that the COVID-19 vaccine will protect her from getting very sick (strongly disagree—strongly agree *)	**1.43**	[1.31,1.56] **
AGYW agrees that the COVID-19 vaccine would be safe for her (strongly disagree—strongly agree *)	**1.51**	[1.41,1.61] **
Most of AGYW’s friends think that getting the COVID-19 vaccine is a good thing (strongly disagree—strongly agree *)	**1.17**	[1.12,1.23] **
Most of AGYW’s family think that getting the COVID-19 vaccine is a good thing (strongly disagree—strongly agree *)	**1.23**	[1.16,1.31] **
**Potential barriers to access**		
The vaccine site is too far away (ref = no)	0.97	[0.90,1.04]
AGYW does not know where to go to get the vaccine (ref = no)	**0.74**	[0.66,0.82] **
The opening times at the vaccine site are not convenient (ref = no)	0.96	[0.88,1.05]
The waiting times at vaccine site are too long (ref = no)	0.98	[0.91,1.05]
It will be expensive for AGYW to get to the vaccine site (ref = no)	**0.81**	[0.69,0.94] *
AGYW cannot go to vaccine site on her own (ref = no)	**0.88**	[0.81,0.95] **
There are not enough vaccines at the vaccine site she wants to go to (ref = no)	0.96	[0.88,1.05]

Bold indicates a barrier or facilitator that has a statistically significant association with motivation or access (*p* < 0.05). * *p* < 0.05, ** *p* < 0.01, strongly disagree—strongly agree * is a five-point Linkert scale with categories strongly disagree, disagree, unsure, agree, strongly agree. AGYW * is adolescent girls and young women. Each individual regression is adjusted by age and SES as priori confounders.

**Table 3:** Final multivariable model of the barriers to and facilitators of partial or full vaccination against COVID-19 (*n* = 2040).

Variable	aRR	95% CI
Age group (years)		
13–17	_	_
18–23	1.10	[0.95,1.27]
Relative socio-economic status group		
Low	_	_
High	1.11	[0.97,1.28]
AGYW * has been tested for COVID-19 (ref = no)	**2.10**	[1.85,2.38] **
Someone in AGYW’s family has died from COVID-19 (ref = no)	1.08	[0.93,1.24]
AGYW worries that she might get COVID-19 (strongly disagree—strongly agree *)	1.00	[0.96,1.04]
AGYW agrees that COVID-19 would be a serious illness for people in her community (strongly disagree—strongly agree *)	1.01	[0.95,1.07]
AGYW is very afraid of the vaccine needle (strongly disagree—strongly agree *)	**0.85**	[0.82,0.88] **
AGYW agrees that the COVID-19 vaccine will protect her from getting very sick (strongly disagree—strongly agree *)	**1.11**	[1.04,1.19] **
AGYW agrees that the COVID-19 vaccine would be safe for her (strongly disagree—strongly agree *)	**1.31**	[1.18,1.44] **
Most of AGYW’s friends think that getting the COVID-19 vaccine is a good thing (strongly disagree—strongly agree *)	1.00	[0.97,1.03]
Most of AGYW’s family think that getting the COVID-19 vaccine is a good thing (strongly disagree—strongly agree *)	1.02	[0.98,1.07]
AGYW does not know where to go to get the vaccine (ref = no)	0.83	[0.68,1.01]
It will be expensive for AGYW to get to the vaccine site (ref = no)	1.16	[0.99,1.36]
AGYW cannot go to vaccine site on her own (ref = no)	0.84	[0.69,1.02]

Bold indicates a barrier or facilitator with a statistically significant association with motivation or access (*p* < 0.05). ** *p* < 0.01, strongly disagree—strongly agree * is a five-point Linkert scale with categories strongly disagree, disagree, unsure, agree, strongly agree. AGYW * is adolescent girls and young women.

## 4. Discussion

This study aimed to measure motivation to take up, access to, and uptake of COVID-19 vaccines, as well as identify barriers to and facilitators of COVID-19 vaccination among AGYW in 14 high schools across two districts in South Africa. The key findings of this study are that 45% of all participants had been vaccinated with at least one dose of a COVID-19 vaccine, and 21% were fully vaccinated. Motivation to take up COVID-19 vaccines was low (49%), while access to COVID-19 vaccines was higher (69%).

By October 2022 (the end of the Imagine Evaluation baseline survey), vaccination coverage with at least one dose of a COVID-19 vaccine among all participants of the Imagine Evaluation (45%) was lower compared to the national average for women aged 35–49 (59%) and 50–59 (65%) in South Africa, as well as the WHO’s target of 70% vaccination coverage by mid-2022 [11,34]. There are several factors which may have contributed to the low vaccination coverage among AGYW in South Africa. First, we will discuss the barriers and facilitators that were identified through our analyses, and then we will discuss other potential contributors.

The findings of this study highlight the key barrier to COVID-19 vaccination as a fear of injections, while facilitators were prior testing for COVID-19, believing that the vaccine would prevent you from getting very sick with COVID-19, and believing that the vaccine is safe. In terms of barriers, fear of injections is a well-known phobia which is particularly prevalent among children and adolescents [35,36]. Fear of injections was also highlighted as a barrier to COVID-19 vaccination in one qualitative study among adolescents and young people in South Africa, as well as a global systematic review of vaccine demand among adolescents [19,20]. With regards to facilitators, it is not surprising that participants who had ever tested for COVID-19 were also more likely to be vaccinated for COVID-19, as both demonstrate a willingness and ability to access COVID-19-related health services. Findings regarding safety and effectiveness of COVID-19 vaccines are supported by findings from a study conducted among adolescents in five sub-Saharan African countries excluding South Africa, which found that concerns about the safety and effectiveness of vaccines was a major barrier to vaccine uptake, except in our study, positive beliefs about safety and effectiveness facilitated vaccine uptake [18]. The delay in vaccine rollout for adolescents and changing recommendations from one-dose of Pfizer in October 2021 to two-doses in December 2021 may have also contributed to concerns about safety and effectiveness among this age group.

Another important finding is that 69% of participants agreed that COVID-19 would be a serious illness for people in their community, but only 36% of participants worried about getting COVID-19 themselves, although neither of these factors were associated with vaccine uptake in the final multivariable model. Nevertheless, we cannot ignore this finding, as it is supported by other evidence which describes the widely held view among adolescents that they were less vulnerable to severe COVID-19 disease. This is reflected in the decision of the South African government (and many other governments) to target the elderly population for the distribution of a limited vaccine supply at the beginning of the pandemic [9,37]. COVID-19 vaccinations were only made available to adolescents in South Africa at the end of the third wave in October 2021. By July 2022, at the end of a miniscule fifth wave, the putative view that the pandemic was behind us in South Africa was well established and reflected in the lifting of most restrictions—including mask wearing in public—and a rapid deceleration of vaccine uptake [38]. This meant that most of the vaccine effort among adolescents took place over a short nine-month period between October 2021 and July 2022 [10].

Despite delays in the commencement of COVID-19 vaccination procurement during 2021 in South Africa, and challenges in delivering vaccines to rural areas in the country, this study found that access to COVID-19 vaccines among schoolgoing AGYW in two study areas with high rates of poverty and unemployment was as high as 69% [39]. Furthermore, SES group was not associated with vaccine uptake, demonstrating that the government was successful in delivering vaccines to the hard-to-reach sites included in this study. By October 2022, vaccine coverage of at least one dose among adolescent girls (43%) and young women aged 18–23 (53%) who participated in this study was also higher than the national average for adolescent girls (38%) and young women aged 18–34 (45%), respectively. This could be because vaccines were successfully provided through school programmes, and all Imagine Evaluation participants were in school, but not all AGYW aged 12–34 in South Africa are in school and they may have faced a multitude of additional challenges in accessing vaccines, including getting to a vaccination site.

Following the logic of the HIV prevention cascade, which describes how motivation and access are required for individuals to take up prevention methods, the findings of this study suggest that interventions to improve uptake of COVID-19 vaccines should aim to increase motivation for COVID-19 vaccines by addressing AGYW’s fear of injections and concerns about the safety and effectiveness of vaccines. A systematic review of 292 reviews and primary studies on the effectiveness of HIV prevention interventions in relation to the HIV prevention cascade suggests that the most effective interventions for demand-side (motivation) barriers to condom use are peer-led information, education, and communication interventions [40]. This is supported by the literature on vaccine hesitancy in South Africa, which also recommends education and communication interventions to improve vaccine uptake and address concerns about their safety and effectiveness [15,17]. These interventions should be provided by different stakeholders involved in the national immunisation programme, including teachers, peer ambassadors, health workers, and community members. The Department of Basic Education may even consider including a section on vaccination in the Life Orientation curriculum in South Africa for routine use or rollout during pandemic situations. Educational interventions should include an exposure-based therapeutic component to desensitise adolescents to vaccination and address any potential needle fears [36]. Health workers should also be trained in distraction techniques to assist in vaccinating individuals with a fear of injections.

A final noteworthy finding of this study is the high percentage of participants that lost family members due to COVID-19 (10%). Of particular concern is the finding that 3% of participants lost grandmothers due to COVID-19. Grandmothers are often the primary caretakers of children and adolescents in South Africa [41]. In addition, 2% of participants report that they had been hospitalised for a COVID-related illness. This is a high rate of hospitalisation for this age group. These findings highlight the trauma that adolescents and young people experienced during the COVID-19 pandemic, which may have impacted participants’ mental health, financial security, and academic performance. These findings are supported by other studies on education and mental health among adolescents during COVID-19 lockdowns, which found that adolescents experienced significant educational disruption and increased stress and anxiety [6,7].

Limitations of this study are that the findings are not generalisable to the two districts in which data were collected, as the 14 study schools may not be representative of all schoolgoing AGYW in these districts. Nevertheless, the results are representative of the 14 study schools. In addition, vaccines were rolled out at all high schools in the country; thus, schoolgoing adolescents in South Africa should all have had access to vaccines even if motivation and uptake differed across the country. Although this study only focused on barriers to and facilitators of vaccine uptake among AGYW, the barriers and facilitators that were identified have already been highlighted as barriers or facilitators among adolescent boys and girls in sub-Saharan Africa and globally. Thus, we do not think that our findings and their implications only apply to AGYW. The findings of the study may also have been influenced by an upward social desirability bias, as participants may have been eager to show their willingness to be vaccinated to ensure their inclusion in future programme interventions. However, since the survey was an anonymised self-assessment, this bias should be minimal. Furthermore, participants were informed that the Imagine Programme would be available to all female learners at their school.

## 5. Conclusions

In conclusion, the WHO and South African government’s goal of 70% vaccination coverage was not achieved in schoolgoing adolescents and young people. As it turns out, there is a high degree of vaccine escape by currently circulating variants of Omicron, and the notion of herd immunity has proven not to be the primary goal of the vaccination programme. Control of severe disease is the stated goal of the vaccination programme, and this has lesser value in this population than older or more vulnerable groups, confirming that the strategy to reach learners later with vaccinations made good sense in hindsight. The rapid expansion of a critical intervention under pandemic conditions, demonstrated by the high level of access achieved in a short period of time as shown in our study, is an important achievement in South Africa. Low uptake is likely linked to the delay in targeting the younger age groups, the shortened time frame in which to scale up the intervention, controversy about the value of targeting the 12–17-year-old population, and as shown in this study, a lack of motivation due to beliefs, attitudes, and fears. A more effective public health education and vaccine promotion programme may have been the missing ingredient in the scale up of vaccinations to schoolgoing learners.

## Figures and Tables

**Table 1:** Socio-demographics, COVID-19 testing and outcomes, and potential barriers and facilitators of motivation to take up and access to COVID-19 vaccines among Imagine Evaluation participants, stratified by age group (*n* = 2377).

Variable	13–17 Age Group (*n* = 1817)		18–23 Age Group (*n* = 560)		Total (*n* = 2377)	
	N (%)	95% CI	N (%)	95% CI	N (%)	95% CI
**Socio-demographics**						
Relatively low socio-economic status	639 (38.5)	[32.9,44.5]	229 (42.8)	[35.0,51.0]	868 (39.6)	[34.2,45.2]
**COVID-19 testing and outcomes**						
AGYW * has been tested for COVID-19	762 (42.0)	[37.4,46.7]	285 (50.9)	[46.2,55.6]	1047 (44.1)	[39.7,48.6]
AGYW thinks she has had COVID-19	209 (11.5)	[9.2,14.3]	61 (10.9)	[8.0,14.7]	270 (11.4)	[9.5,13.6]
Someone in AGYW’s home has tested positive for COVID-19	513 (28.3)	[24.3,32.6]	119 (21.3)	[17.5,25.5]	632 (26.6)	[23.2,30.4]
AGYW went to hospital for COVID-19	32 (1.8)	[1.2,2.6]	8 (1.4)	[0.6,3.4]	40 (1.7)	[1.2,2.4]
Someone in AGYW’s family went to hospital for COVID-19	**345 (19.2)**	**[17.0,21.6]**	**68 (12.3)**	**[9.5,15.8]**	**413 (17.6)**	**[15.8,19.5]**
Someone in AGYW’s family has died from COVID-19	188 (10.4)	[8.7,12.3]	60 (10.7)	[7.2,15.6]	248 (10.4)	[9.3,11.7]
**Potential barriers and facilitators of motivation**						
AGYW worries that she might get COVID-19	653 (36.0)	[33.4,38.6]	204 (36.4)	[31.6,41.6]	857 (36.1)	[33.4,38.9]
AGYW agrees that COVID-19 would be a serious illness for people in her community	1244 (68.5)	[65.9,71.1]	392 (70.0)	[64.2,75.2]	1636 (68.9)	[66.6,71.1]
AGYW is very afraid of the vaccine needle	1052 (58.0)	[53.4,62.4]	322 (57.5)	[52.5,62.3]	1374 (57.9)	[54.0,61.6]
AGYW agrees that the COVID-19 vaccine will protect her from getting very sick	1029 (56.7)	[53.6,59.8]	356 (63.6)	[56.7,69.9]	1385 (58.3)	[54.7,61.9]
AGYW agrees that the COVID-19 vaccine would be safe for her	924 (50.9)	[46.4,55.4]	324 (57.9)	[52.0,63.5]	1248 (52.5)	[48.3,56.8]
Most of AGYW’s friends think that getting the COVID-19 vaccine is a good thing	934 (51.5)	[48.1,54.8]	298 (53.2)	[45.4,60.8]	1232 (51.9)	[48.1,55.6]
Most of AGYW’s family think that getting the COVID-19 vaccine is a good thing	1058 (58.3)	[56.5,60.1]	322 (57.5)	[50.0,64.7]	1380 (58.1)	[55.7,60.5]
**Potential barriers and facilitators of access**						
The vaccine site is too far away	480 (26.4)	[24.1,28.9]	174 (31.1)	[27.5,34.9]	654 (27.5)	[25.3,29.9]
AGYW does not know where to go to get the vaccine	265 (14.6)	[12.5,17.0]	78 (13.9)	[10.4,18.5]	343 (14.4)	[12.3,16.8]
The opening times at the vaccine site are not convenient	187 (10.3)	[8.9,11.9]	55 (9.8)	[7.9,12.1]	242 (10.2)	[9.1,11.4]
The waiting times at vaccine site are too long	404 (22.3)	[20.3,24.3]	131 (23.4)	[17.8,30.1]	535 (22.5)	[20.4,24.8]
It will be expensive for AGYW to get to the vaccine site	89 (4.9)	[3.9,6.1]	23 (4.1)	[2.4,7.0]	112 (4.7)	[3.6,6.1]
AGYW cannot go to vaccine site on her own	**333 (18.3)**	**[15.8,21.2]**	**53 (9.5)**	**[6.4,13.8]**	**386 (16.3)**	**[13.6,19.3]**
There are not enough vaccines at the vaccine site she wants to go to	121 (6.7)	[5.0,8.8]	30 (5.4)	[3.5,8.1]	151 (6.4)	[5.0,8.1]

Bold indicates a statistically significant difference between age groups. There are a maximum of two observations missing per variable except for relative socio-economic status, which is missing in 184 observations. AGYW * is adolescent girls and young women.

## Data Availability

Data is available on request.
